# Development of Adjustable Parallel Helmholtz Acoustic Metamaterial for Broad Low-Frequency Sound Absorption Band

**DOI:** 10.3390/ma15175938

**Published:** 2022-08-27

**Authors:** Xiaocui Yang, Fei Yang, Xinmin Shen, Enshuai Wang, Xiaonan Zhang, Cheng Shen, Wenqiang Peng

**Affiliations:** 1 Engineering Training Center, Nanjing Vocational University of Industry Technology, Nanjing 210023, China; 2 MIIT Key Laboratory of Multifunctional Lightweight Materials and Structures (MLMS), Nanjing University of Aeronautics and Astronautics, Nanjing 210016, China; 3 College of Field Engineering, Army Engineering University of PLA, Nanjing 210007, China; 4 College of Aerospace Science and Engineering, National University of Defense Technology, Changsha 410073, China

**Keywords:** adjustable parallel helmholtz acoustic metamaterial, tunable chamber, low-frequency noise control, broadband, transfer function tube measurement, finite element simulation

## Abstract

For the common difficulties of noise control in a low frequency region, an adjustable parallel Helmholtz acoustic metamaterial (APH-AM) was developed to gain broad sound absorption band by introducing multiple resonant chambers to enlarge the absorption bandwidth and tuning length of rear cavity for each chamber. Based on the coupling analysis of double resonators, the generation mechanism of broad sound absorption by adjusting the structural parameters was analyzed, which provided a foundation for the development of APH-AM with tunable chambers. Different from other optimization designs by theoretical modeling or finite element simulation, the adjustment of sound absorption performance for the proposed APH-AM could be directly conducted in transfer function tube measurement by changing the length of rear cavity for each chamber. According to optimization process of APH-AM, The target for all sound absorption coefficients above 0.9 was achieved in 602–1287 Hz with normal incidence and that for all sound absorption coefficients above 0.85 was obtained in 618–1482 Hz. The distributions of sound pressure for peak absorption frequency points were obtained in the finite element simulation, which could exhibit its sound absorption mechanism. Meanwhile, the sound absorption performance of the APH-AM with larger length of the aperture and that with smaller diameter of the aperture were discussed by finite element simulation, which could further show the potential of APH-AM in the low-frequency sound absorption. The proposed APH-AM could improve efficiency and accuracy in adjusting sound absorption performance purposefully, which would promote its practical application in low-frequency noise control.

## 1. Introduction

Noise pollution has been considered as one of the most important pollution sources to human lives, which disturbs our daily life and harms our health; especially noise in the low frequency region, which is difficult to dissipate and attenuate in the spreading process for its high penetrability with large wavelength [1,2]. Most noise is generated by the equipment, such as construction equipment, vehicles, machine tools and household appliances [3,4]. Taking into account that reducing the generated noise through optimizing the mechanical structure or assembly process of equipment is difficult to conduct, the application of sound absorbing materials or structures in the noise propagation path is treated as a feasible and practicable method for noise control [5,6]. Relative to traditional sound absorbing materials, such as porous material [7] and microperforated panels [8], the acoustic metamaterial can obtain a higher sound dissipation in the low frequency region with a relatively smaller occupied space [9]. At present, many kinds of acoustic metamaterials have been developed, which exhibit their advantages in low frequency noise control.

The sound absorption performance of acoustic metamaterial is mainly decided by its structural parameters, which can obtain a certain frequency bandwidth with a certain size [10,11,12,13,14]. Wu et al. had proposed an acoustic metamaterial for the low frequency absorption through the combination of three nested square split tubes with the inverted opening direction, and three absorption peaks at the 230 Hz, 320 Hz, and 500 Hz were obtained by adjusting the geometric parameters [10]. The microperforated acoustic metamaterial with honeycomb-corrugation hybrid core was developed by Zhang et al. [11] to achieve the broadband low frequency noise control, and the influence of its key geometrical parameters on the sound absorption was systematically investigated, such as the upper facesheet thickness, perforation diameter and corrugated plate thickness. Ma et al. [12] had developed a metamaterial design method that used soft matter for constructing a unique soft acoustic boundary to effectively improve the sound absorption performance. Yang and Sheng [13] had investigated the sound absorption structures from porous media to acoustic metamaterials, and absorption performance of each kind of absorber was summarized. A novel acoustic metamaterial structure made up of both resonator and multiple channels was developed by Xu et al. [14] to insulate low-frequency broadband noises, and its low-frequency broadband sound insulation performance could be improved by changing structural parameters and materials. Therefore, structural parameters of the acoustic metamaterial must be strictly selected to achieve an excellent sound absorption property.

In order to obtain the desired sound absorption performance, it is essential to adjust structural parameters of the acoustic metamaterial [15,16,17,18,19]. Li et al. [15] had proposed the composite structure composed of porous-material layer mosaicked with a perforated resonator and its structural parameters were optimized to enhance the low-frequency sound absorption of the porous layer. An optimization methodology was proposed by Lee et al. [16] to develop a more compact local resonant sonic crystals window, and three optimal sets of design parameters on the corresponding interested frequencies of 630 Hz, 800 Hz and 1000 Hz were obtained. Ciaburro et al. [17] proposed a novel layered membrane metamaterial based on three layers of the reused PVC membranes and a reused metal washer, and different configurations were analyzed by changing the number of masses attached to each layer and the geometry of their position. A Helmholtz-type phononic crystal with adjustable cavity structure and labyrinth tubes was designed by Han et al. [18], and multiple resonant band gaps could be connected by adjusting the structural layout of the cavity through the telescopic screw, so as to achieve the purpose of widening the band gap and the active control of environmental noise. Wang et al. [19] had developed an acoustic metamaterial absorber of parallel-connection square Helmholtz resonators, and the average actual sound absorption coefficients of these three optimized metamaterial cells were 0.9271 in the [700 Hz, 1000 Hz] with a total size of 30 mm, 0.9157 in the [600 Hz, 900 Hz] with a total size of 40 mm, and 0.9259 in the [500 Hz, 800 Hz] with a total size of 50 mm, respectively. It has been proved that optimization design is essential and effective method to achieve low frequency noise control with broadband for the acoustic metamaterial.

However, it can be found that most of the present optimizations for the acoustic metamaterial are based on theoretical modeling [20,21,22] or finite element simulation [23,24]. Gritsenko and Paoli [20] had proposed a novel theoretical approach to characterize bubble-based metamaterials based on the findings for a single bubble trapped in circular cavity modeled as a thin clamped plate and further conducted the theoretical optimization of the trapped-bubble-based acoustic metamaterial performance. The receptance functions of metamaterial beams with various resonator connection architectures were derived by Wei et al. [21] according to the transfer matrix model and further be verified through finite element model results. Palma and Burghignoli [22] had conducted the theoretical link between two widely used metamaterial models of transformation acoustics and generalized Snell’s Law, and it was exploited to perform an optimal design of phase-graded metasurface acoustic lining of a 2-D duct in presence of flow. Tang et al. [23] had built the finite element model of the perforated honeycomb-corrugation hybrid to analyze its absorption properties and research viscous-thermal energy dissipation. The anti-tetra-chiral auxetic sandwich panel and its auxetic core were studied through using full-scale finite element simulation approach by Hosseinkhani et al. [24], and an equivalent homogenized model was employed to reduce the computational time of optimization problem. As is known to all, the normal constructed theoretical sound absorption model of acoustic metamaterial or metastructure is imprecise for ineluctable simplifications, approximations, hypothesis and abbreviations. Although the acoustic finite element simulation method can achieve a better prediction accuracy for sound absorption coefficients of the acoustic metamaterial, it still has deviation relative to the actual data and the simulation takes a long time. Thus, the adjustable acoustic metamaterial was developed in this research, which aimed to conduct the adjustment of sound absorption property directly in experimental process.

The acoustic metamaterial of parallel connection of Helmholtz resonators had been widely applied in the field of reduction of low frequency noise [25,26,27,28,29], and its sound absorption performance could be easily altered through adjusting the structural parameters of diameter and length of the aperture, thickness and size of the cavity, perforation ratio and number of the chamber. Thus, the acoustic metamaterial of the parallel connection of multiple Helmholtz resonators with adjustable chamber was optimized as investigation target in this study, which aimed to obtain excellent sound absorption performance in the low frequency region for the certain application requirement. The proposed adjustable parallel Helmholtz acoustic metamaterial (abbreviated as the APH-AM) was developed by introducing the multiple resonant chambers and tuning the length of rear cavity for each chamber. It should be noted that the tuning mechanism required manual mechanical intervention and it was not an automatic process, which was quite different from the electronically programmable metasurfaces [30,31,32,33,34,35]. The sound field minimization through in situ engineered destructive interferences could maximize/minimize the sound field inside a room at a desired point for a static single-frequency [30], a static multi-frequency or a transient multi-frequency field [31], which could achieve ultrabroadband absorber in microwave domain [33]. The selection of tuning mechanism in this study had taken into account the occupied space and manufacturing cost simultaneously, because the difficulty level in adjusting different kind of structural parameter for the APH-AM was obviously dissimilar. Therefore, the length of rear cavity for each chamber was selected as the tunable parameter and the other parameters were selected by taking the manufacturing cost into account, which aimed to promote the practical application of APH-AM.

In this study, based on the coupling analysis of double resonators by the finite element simulation, the generation mechanism of broad sound absorption by adjusting the structural parameters of the APH-AM was analyzed, which provided the foundation to achieve the broad sound absorption bandwidth. Different from other optimization design by the theoretical modeling or finite element simulation, the adjustment of sound absorption performance for the proposed APH-AM could be directly conducted in transfer function tube measurement by changing the length of rear cavity for each chamber, which was realized by controlling the slider moving along the 4 guide rails in each chamber. Taking into consideration that the given target sound absorption performances for certain application conditions, the proposed APH-AM with tunable cavities was detected repeatedly with different parameters of cavities based on the transfer function method with normal incidence, which aimed to achieve fine sound absorption performance in the target frequency region. Afterward, the distributions of sound pressure for these peak absorption frequency points of the optimized APH-AM sample were obtained by the finite element simulation, which can exhibit sound absorption mechanism. Meanwhile, the sound absorption performance of the APH-AM with larger length of the aperture and that with smaller diameter of the aperture were discussed by finite element simulation, which could further show the potential of APH-AM in the low-frequency sound absorption.

## 2. Materials and Design

### 2.1. Structural Design

**Figure 1:**
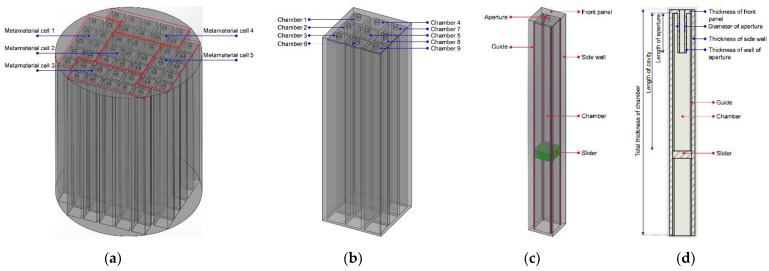
A schematic diagram of the APH-AM structure. (**a**) 3D model of the whole structure; (**b**) 3D model of a metamaterial cell; (**c**) 3D model of a single adjustable chamber; (**d**) 2D sketch and parameters of a single adjustable chamber.

A schematic diagram of the proposed APH-AM structure is exhibited in Figure 1. Taking into consideration that the required size of tested sample in the transfer function tube measurement is Φ100 mm, the APH-AM structure is limited in the Φ100 mm, as shown in the Figure 1a. Meanwhile, the basic metamaterial cell utilized in this study composed of 9 parallel Helmholtz resonators with the side length of 10 mm, as shown in the Figure 1b. Thus, the Helmholtz resonators in the Figure 1a included 5 basic metamaterial cells, as shown in the Figure 1a. Furthermore, the length of rear cavity for each chamber was controlled through moving the slider along the 4 guides in the chamber, as shown in the Figure 1c, and the slider was driven by the linear actuator. In order to avoid leakage noise in the detection process and reduce the friction between the slider and guide, the silicone oil was applied on the 4 guides. Moreover, thickness of side wall for each chamber was selected as 1.8 mm and that of front panel was set as 2 mm, as shown in the Figure 1d, which took structural stability and reliability of APH-AM into account. By controlling the slider moving along the guides in each chamber, the proposed APH-AM was realized.

### 2.2. Theoretical Analysis

As mentioned in Section 1, the sound absorption performance of parallel connection of Helmholtz resonators could be adjusted by changing the structural parameters of diameter and length of the aperture, thickness and size of the cavity, perforation ratio and number of the chamber [36,37,38,39,40]. Gao et al. [36] had design a slit-type unit cell as a practical implementation of the coupling modulation of resonance energy leakage and loss in ventilated metamaterials and further analytically proved its potential to obtain the desired leakage and loss factors simultaneously by properly adjusting the structural parameters. A genetic and general nonlinear constrained algorithm was utilized by Meng et al. [37] to enhance the low-frequency underwater sound absorption of an acoustic metamaterial slab with several layers, and both the physical and structural parameters of the acoustic metamaterial slab were optimized to enlarge the absorption band. Gaafer [38] had used finite element simulations via COMSOL Multiphysics software to theoretical measurement in impedance tube and showed the influence of structural parameters for a modulus-near-zero (MNZ) metamaterial immersed in air or water with a change in slit width part. Based on the multiphysics field coupling method and thermoviscous acoustic theory, the effect of structural parameter changes on the sound absorption performance of metamaterial structure was investigated by Cui et al. [39] through using finite element analysis. Du et al. [40] had summarized the structural design of typical acoustic metamaterials of membrane, plates, Helmholtz cavities, and coupling structures from the structural design perspective, which provided guidance for the potential application of acoustic metamaterials in engineering practice.

Therefore, except the number of chambers, influence of other 5 parameters to sound absorption performance of the double resonators was studied by finite element simulation, which would provide explanation for development and design of the proposed APH-AM in this research according to the guidance obtained in the relevant literatures [36,37,38,39,40].

#### 2.2.1. Finite Element Simulation Model

**Figure 2:**
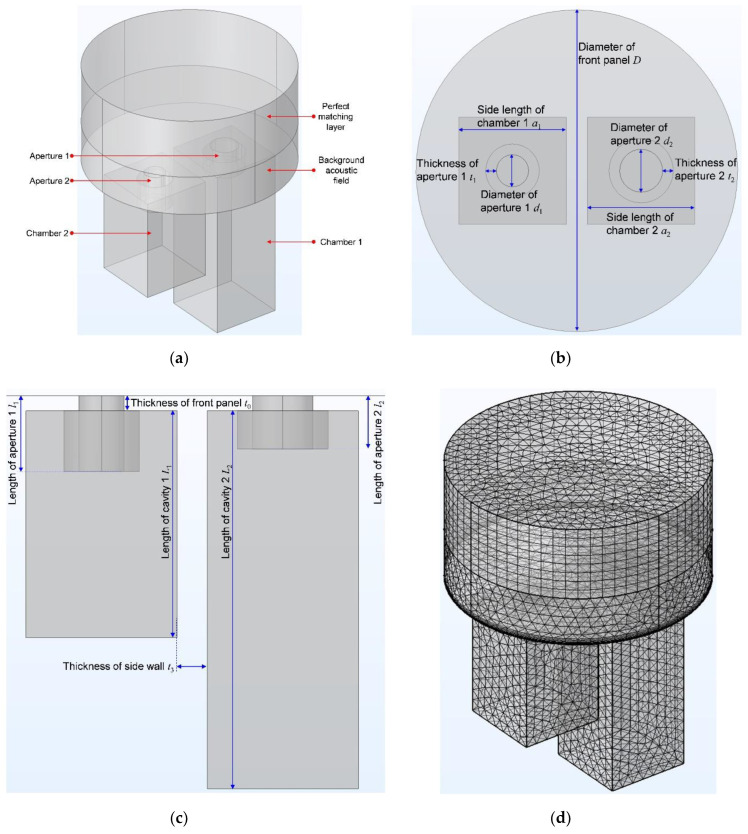
A 3-dimensional finite element simulation model for the double resonators. (**a**) 3D structure of the whole model; (**b**) The vertical view of model with the relevant parameters; (**c**) The front view of model with the relevant parameters; (**d**) The finite element simulation model with the gridded mesh.

A 3–dimensional finite element simulation model for the double resonators was constructed, as shown in the Figure 2, which included the perfect matching layer, background acoustic field and the two resonators. Each resonator had an aperture and a chamber, as shown in the Figure 2a. Diameter of the front panel *D* was equal to diameter of the perfect matching layer and that of the background acoustic field, which was used to calculate the perforation ratio for each resonator. For each resonator, the parameters consisted of thickness, diameter and length of the aperture *t*_i_, *d*_i_ and *l*_i_ (i = 1, 2); side length of the chamber *a*_i_; thickness of the front panel *t*_0_; thickness of the side wall *t*_3_; length of the cavity *L*_i_, as shown in the Figure 2b,c. Some of the parameters had few influences to sound absorption performance of the double resonators, such as *t*_0_, *t*_1_, *t*_2_, and *t*_3_, thus there values are set as 2 mm, 1 mm, 1 mm and 1.8 mm respectively, which had taken manufacturing cost, structural compactness and mechanical stability into consideration. The other parameters were investigated their influences to sound absorption performance of the double resonators in the following parts, which included perforation ratio, diameter of the aperture, length of the aperture, length of the cavity and size of the cavity successively. Through gridding the finite element simulation model, as shown in the Figure 2d, sound absorption coefficients of the double resonators could be obtained in simulation. The parameters for the gridded mesh consisted of size of the maximum cell 2.87 mm, size of the minimum cell 0.123 mm, maximum unit growth rate 1.35, curvature factor 0.3, and resolution of the narrow region 0.85. In order to increase the simulation accuracy, the boundary layers were refined with the stretching factor 1.2 and regulatory factor of their thicknesses 1. Meanwhile, the plane wave with the amplitude 1 was utilized in the background acoustic field as the incident sound wave, and the perfect matching layer was used to simulate the absorption of sound waves in the process of propagation away from the sound source. Moreover, side boundaries of the perfect matching layer and those of the background acoustic field were set as slip boundaries, which would not generate the thermal viscous effect. For the other side boundaries in the Figure 2d, they were set as nonslip boundaries. All the boundaries were under the isothermal condition.

The direct linear solver of PARDISO was selected as steady-state solver in the finite element simulation procedure, which could utilize the shared memory parallel processing to improve the computational efficiency. In order to avoid the gridded mesh too small, the pre-sorting algorithm based on space filling curve was utilized in the PARDISO. The parameters of the parallel direct sparse solver for cluster computing were memory fraction outside the core 0.99, total memory usage ratio 0.8, and internal memory usage factor 3. Meanwhile, the error estimation factor was set as 1, and the parameters for the iterative refinement were the maximum mesh refinement 15 and error rate range 0.5. For the other setups in the finite element simulation process, they were set as normal selections in the thermoviscous acoustics modular in COMSOL Multiphysics software.

The basic equations for the finite element model were shown in the Equations (1)–(3), which corresponded to the acoustic pressure field, acoustic velocity field and acoustic temperature field respectively.

(1) $$p_{t}=p+p_{b}$$

(2) $$u_{t}=u+u_{b}$$

(3) $$T_{t}=T+T_{b}$$

The value of the background acoustic field variables *p_b_*, *u_b_*, and *T_b_* could be calculated by the Equations (4)–(6) respectively. In the Equations (4)–(6), the parameter *k_b_* and *n_k_* could be derived by the Equations (7) and (8), respectively, and the parameter *b_tv_* in the Equation (7) could be calculated by the Equation (9). Definitions of these parameters were summarized in the COMSOL Multiphysics software. It should be noted that the *e_k_* represented the direction of the incident wave. For the normal incidence, the *e_k_* should be [0, 0, −1]. For the oblique incidence with an oblique angle of *θ*, the *e_k_* should be [sin *θ*, 0, −cos *θ*].

(4) $$p_{b}=\left|p_{b}\right|e^{-ik_{b}n_{k}x}$$

(5) $$u_{b}=\frac{\omega \left(\beta_{T}p_{b}-\alpha_{p}T_{b}\right)}{k_{b}}n_{k}$$

(6) $$T_{b}=\frac{i\omega \alpha_{p}T_{0}}{i\omega \rho_{0}C_{p}+kk_{b}^{2}}p_{b}$$

(7) $$k_{b}=\frac{\omega }{c}{\left(1+\frac{i\omega b_{tv}}{\rho_{0}c^{2}}\right)}^{-1/2}$$

(8) $$n_{k}=\frac{e_{k}}{\left|e_{k}\right|}$$

(9) $$b_{tv}=\frac{4}{3}\mu +\mu_{B}+\frac{\left(\gamma -1\right)k}{C_{p}}$$

Through calculating the acoustic pressure field, acoustic velocity field and acoustic temperature field in each aperture and chamber for the double resonators, its sound absorption coefficients could be derived. The finite element simulation provided foundation to achieve broad sound absorption bandwidth for APH-AM. According to the normal parameters for the common Helmholtz resonator and taking the manufacturing cost and difficulty level into account as well, the reference values for the investigated parameters of perforation ratio, diameter of the aperture, length of the aperture, length of the cavity and size of the cavity were selected as 0.7%, 2.7 mm, 6 mm, 50 mm and 10 mm, respectively.

#### 2.2.2. Perforation Ratio

There was a dimensional limit for each resonator in the parallel connection of multiple resonators, and excellent sound absorption performance could be obtained only when the size of front panel was controlled in a reasonable range. For the classical microperforated panel absorber, perforation ratio was defined as ratio between the area of hole and that of the whole panel. According to this definition, perforation ratio *σ* was defined as ratio between the area of aperture and that of the front panel in the Figure 2b, which could be calculated by the Equation (10). Here, n was the number of aperture with the same diameter.

(10) $$\sigma =n\cdot \frac{\pi {\left(d/2\right)}^{2}}{\pi {\left(D/2\right)}^{2}}$$

**Figure 3:**
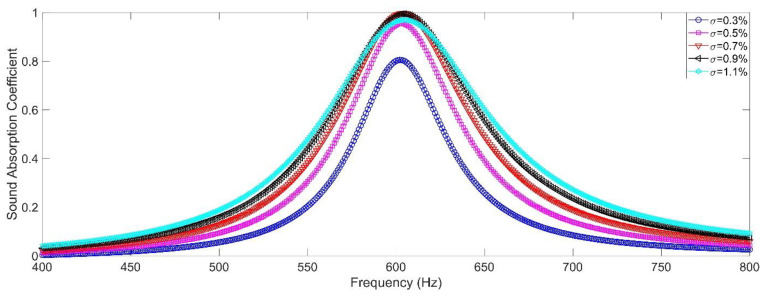
The influence of the perforation ratio to sound absorption performance of the double resonators in simulation.

Influence of the perforation ratio *σ* to sound absorption performance of the double resonators was investigated by changing diameter of the front panel *D*, and the studied perforation ratio *σ* was 0.3%, 0.5%, 0.7%, 0.9% and 1.1%, respectively, as shown in the Figure 3. The other parameters for the double resonators were set as *d*_1_ = *d*_2_ = 2.7 mm, *l*_1_ = *l*_2_ = 6.0 mm, *a*_1_ = *a*_2_ = 10 mm, and *L*_1_ = *L*_2_ = 50 mm. It could be observed that the peak sound absorption coefficient *α*_max_ was 0.8052, 0.9538, 0.9972, 0.9942 and 0.9695 along with increase of perforation ratio *σ* from 0.3% to 1.1%, and each resonator frequency *f*_0_ was around the 604 Hz. The perforation ratio had little influence to the resonator frequency *f*_0_ and it significantly affected the peak sound absorption coefficient *α*_max_. The best peak sound absorption coefficient *α*_max_ was obtained when the perforation ratio *σ* was around 0.7%, which could gain perfect absorption with sound absorption coefficient close to 1. Thus, the perforation ratio should be strictly controlled in the reasonable range, otherwise the peak sound absorption coefficient *α*_max_ would decrease when *σ* was too large or too small. It could be found from Figure 2a,b that all the chambers should be in the range of front panel and the aperture should be in the region of corresponding chamber, which indicated that number of the introduced chambers was limited. For a given front panel, increase of the number of chambers would result in decrease of its area inevitably, and the perforation ratio *σ* was restricted by limit of diameter of the aperture in the chamber.

#### 2.2.3. Diameter of the Aperture

The influence of diameter of the aperture was investigated, and the diameter of the aperture 2 *d*_2_ was selected as the studied variable. Its value was selected as 1.7 mm, 2.2 mm, 2.7 mm, 3.2 mm and 3.7 mm, respectively. Save for the diameter of the aperture 2 *d*_2_, the other parameters were selected as *d*_1_ = 2.7 mm, *l*_1_ = *l*_2_ = 6 mm, *L*_1_ = *L*_2_ = 50 mm, *a*_1_ = *a*_2_ = 10 mm, and *σ*_2_ = 0.7% (which indicated that the corresponding diameter of the front panel *D* was 20.32 mm, 26.29 mm, 32.27 mm, 38.25 mm and 44.22 mm, respectively, along with increase of *d*_2_ from 1.7 mm to 3.7 mm with the interval of 0.5 mm). Therefore, the corresponding perforation ratio for resonator 1 *σ*_1_ was 1.76%, 1.05%, 0.70%, 0.50% and 0.37% respectively, which could be calculated by the Equation (1).

**Figure 4:**
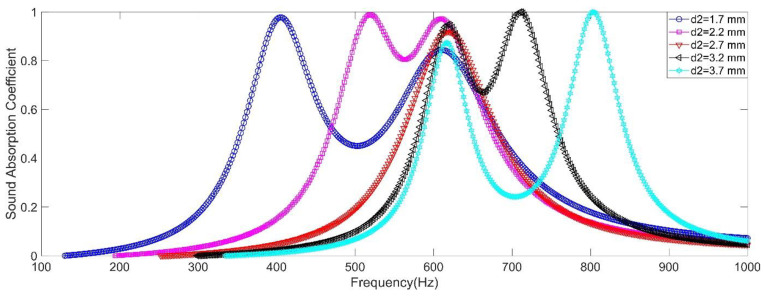
The influence of the diameter of the aperture to sound absorption performance of the double resonators in simulation.

The influence of diameter of the aperture to sound absorption performance of the double resonators in simulation was shown in the Figure 4, and summary of peak sound absorption coefficient *α*_max_ and resonance frequency *f*_0_ was shown in the Table 1. It could be found that except the condition of *d*_1_ = *d*_2_ = 2.7 mm, all the other 4 conditions could obtain the double sound absorption peaks. The first absorption peak was generated by the resonator 1, and its resonator frequency *f*_0_ had little variation. However, its peak sound absorption coefficient *α*_max_ was better relatively when the interval between *d*_1_ and *d*_2_ was smaller. The major reason for this phenomenon was that the perforation ratio *σ*_1_ was different when diameter of the front panel *D* was changed to keep *σ*_2_ = 0.7%, which resulted in mismatch of the acoustic impedance in the resonator 1. Meanwhile, the second absorption peak was generated by the resonator 2 and its resonator frequency *f*_0_ moved to the high frequency direction along with increase of *d*_2_. However, the peak sound absorption coefficient *α*_max_ had few undulation, because the perforation ratio *σ*_2_ was kept constant of 0.7%. Moreover, there was only one resonator frequency for *d*_1_ = *d*_2_ = 2.7 mm, because the structural parameters of two resonators were same. It could be found the peak sound absorption coefficient *α*_max_ was 0.92 at this condition, which was not close to 1. The reason for this phenomenon was that the actual perforation ratio was 1.4% instead of 0.7% when *d*_1_ = *d*_2_ = 2.7 mm, which could be judged from Equation (1) with n = 2 for this condition. It could be concluded that the generation of resonators with different diameter of the aperture could enlarge the absorption bandwidth, and the interval should be controlled reasonably to obtain an excellent sound absorption performance. However, the change of diameter of the aperture was difficult to realize for an already prepared acoustic metamaterial, especially that diameter of the aperture in the acoustic metamaterial is in the mm level normally.

#### 2.2.4. Length of the Aperture

**Figure 5:**
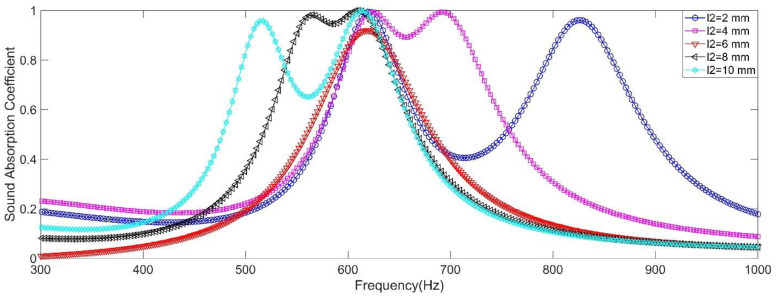
The influence of length of the aperture to sound absorption performance of the double resonators in simulation.

The influence of length of the aperture was investigated and the length of the aperture 2 *l*_2_ was selected as the studied variable. Its value was selected as 2 mm, 4 mm, 6 mm, 8 mm and 10 mm. Except length of the aperture 2 *l*_2_, the other parameters were set as *d*_1_ = *d*_2_ = 2.7 mm, *l*_1_ = 6 mm, *L*_1_ = *L*_2_ = 50 mm, *a*_1_ = *a*_2_ = 10 mm, and *σ*_1_ = *σ*_2_ = 0.7% (which indicated that diameter of the front panel *D* was 32.27 mm). The influence of length of the aperture to sound absorption performance of the double resonators in the simulation was shown in the Figure 5, and the summary of peak sound absorption coefficient *α*_max_ and resonance frequency *f*_0_ was shown in the Table 2. It could be found that introduction of two resonators with different length of the aperture could generate two absorption peaks. The differences between the resonance frequencies of two absorption peaks were 207 Hz, 66 Hz, 0 Hz, 46 Hz and 96 Hz with the *l*_2_ changing from 2 mm to 10 mm with the interval of 2 mm, and all the peak sound absorption coefficients *α*_max_ were above 0.92. Furthermore, the resonance frequency *f*_0_ of resonator 1 was around 618 Hz, and the tiny undulation from 610 Hz to 625 Hz was generated by the coupling effect of resonator 2. Moreover, it could be observed that for the conditions of *l*_2_ = 4 mm and *l*_2_ = 8 mm, the resonance frequency *f*_0_ of two resonators was close to each other and the sound absorption coefficients between the two resonance frequencies were also above 0.9, which kept the excellent sound absorption performance and enlarged the sound absorption bandwidth simultaneously. Through introducing multiple resonators with different length of the apertures, the multiple sound absorption peaks could be generated and the peak sound absorption coefficient was almost not affected. But the aperture was also fixed in the acoustic metamaterial. Thus, similar with diameter of the aperture, length of the aperture was also difficult to adjust for an already prepared acoustic metamaterial.

#### 2.2.5. Length of the Cavity

**Figure 6:**
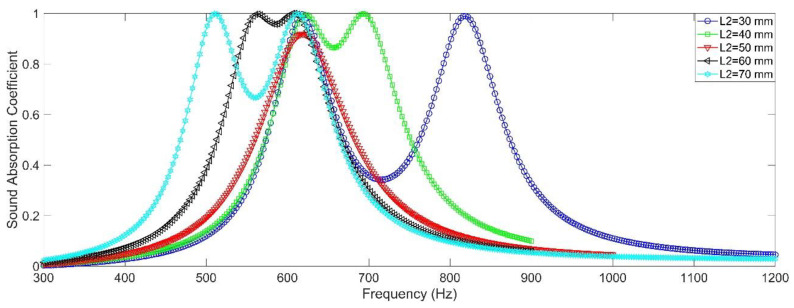
The influence of the length of the cavity to sound absorption performance of the double resonators in simulation.

The influence of length of the cavity was investigated and the length of cavity 2 *L*_2_ was selected as the analyzed variable. Its value was selected as 30 mm, 40 mm, 50 mm, 60 mm and 60 mm. Except the length of the cavity 2 *L*_2_, the other parameters were set as *d*_1_ = *d*_2_ = 2.7 mm, *l*_1_ = *l*_2_ = 6 mm, *L*_1_ = 50 mm, *a*_1_ = *a*_2_ = 10 mm, and *σ*_1_ = *σ*_2_ = 0.7% (which indicated that diameter of the front panel *D* was 32.27 mm). The influence of length of the cavity to sound absorption performance of the double resonators in simulation was shown in the Figure 6, and summary of peak sound absorption coefficient *α*_max_ and resonance frequency *f*_0_ was shown in the Table 3. It could be found that except the condition of *L*_1_ = *L*_2_ = 50 mm, all the other 4 conditions could obtain the double sound absorption peaks, and the differences between resonance frequencies *f*_0_ of the two resonators for each condition were 201 Hz, 72 Hz, 51 Hz and 105 Hz respectively. Meanwhile, all the peak sound absorption coefficients *α*_max_ in these 4 conditions were above 0.99, which exhibited near perfect absorption effect. Moreover, the resonance frequencies *f*_0_ of resonator 1 in all the 5 conditions were kept around 618 Hz, which was almost not affected by the change of length of the cavity for the resonator 2. Furthermore, the resonance frequencies *f*_0_ of resonator 2 moved to the low frequency direction along with the increase of length of its cavity, which was easy to realize adjustment by moving the back panel in the chamber relative to length or diameter of the aperture and other parameters. Therefore, length of the cavity was selected as the adjustable variable for the proposed APH-AM in this research, which was same with the selection in some literatures [41,42,43,44]. The absorption peak was controlled through adjusting the geometric parameters of the Helmholtz resonator with embedded apertures by Zhang et al. [41], and the absorption bandwidth could also be flexibly adjusted with a fixed thickness. Peng et al. [43] had optimized the second cavity in the dual Helmholtz resonators, which could significantly increase the generated electric voltage up to 400%. The cavity-based design of resonant cavity-based piezoelectric micromachined ultrasonic transducers was developed by Xu et al. [44], which aimed to improve its performance.

#### 2.2.6. Size of the Cavity

**Figure 7:**
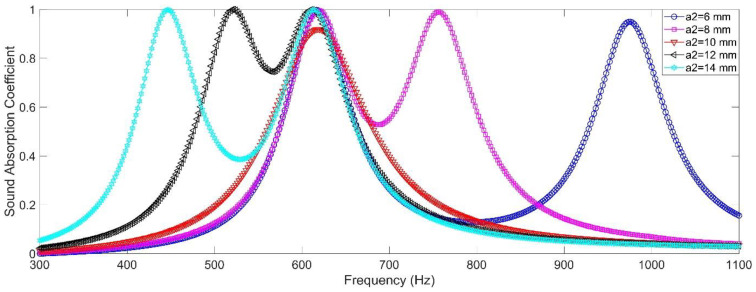
The influence of the size of the cavity to sound absorption performance of the double resonators in simulation.

Besides diameter and length of the aperture and length of the cavity, influence of size of the cavity was investigated and the side length of chamber 2 *a*_2_ was selected as the analyzed variable. Its value was selected as 6 mm, 8 mm, 10 mm, 12 mm and 14 mm. Except side length of the chamber 2 *a*_2_, the other parameters were set as *d*_1_ = *d*_2_ = 2.7 mm, *l*_1_ = *l*_2_ = 6 mm, *L*_1_ = *L*_2_ = 50 mm, *a*_1_ = 10 mm, and *σ*_1_ = *σ*_2_ = 0.7%. Influence of side length of the chamber to sound absorption performance of the double resonators in simulation was shown in the Figure 7, and the summary of peak sound absorption coefficient *α*_max_ and resonance frequency *f*_0_ was shown in the Table 4. It could be found that except the condition of *a*_1_ = *a*_2_ = 10 mm, all the other 4 conditions could also obtain the double sound absorption peaks, and the differences between resonance frequencies *f*_0_ of the two resonators were 356 Hz, 136 Hz, 90 Hz and 168 Hz respectively. Meanwhile, all the peak sound absorption coefficients *α*_max_ in these 4 conditions were above 0.95, which exhibited near perfect absorption effect. Moreover, it could be observed that the offset of resonance frequency *f*_0_ was larger in the high frequency range relative to that in the low frequency range along with the increase of side length of chamber 2, which indicated that the change of resonance frequency *f*_0_ resulted from adjustment of the size of cavity was more sensitive in high frequency region relative to that in the low frequency area. However, adjustment of the size of cavity was also difficult to realize for an already prepared acoustic metamaterial, because the change of side length of one chamber would inevitably affect that of the neighboring chambers around it, which decreased relative independence of each chamber and increased the difficulty of adjustment to achieve an excellent sound absorption performance.

According to the analysis of influence of structural parameters to sound absorption performance of double resonators and the similar research in literatures [41,42,43,44], it could be concluded that length of the cavity was the most suitable parameter to be the adjustable variable, which was easy to realize the adjustment for an already prepared acoustic metamaterial and it did not influence relative independence of each chamber. Thus, this analysis provided theoretical foundation and basis for the proposed APH-AM in this research.

### 2.3. Optimization Process

It could be deduced that there would be 9 absorption peaks for the proposed APH-AM with variable length of the cavity in this research. Taking into consideration that the permitted length of the tested sample in the utilized transfer function tube measurement is no more than 100 mm and the permissible space to install the acoustic metamaterials for certain application conditions in the factory workshop, the range of optional value for length of the cavity in each chamber was limited as 0–90 mm.

In order to avoid the duplication and same value for different chambers, it was set that length of the cavity for chamber 1 *L*_1_ was larger than that for the chamber 2 *L*_2_ in the Figure 2b, and the similar principle was suitable for the other chambers, as shown in the Equation (11), which indicated that length of the cavity decreased sequentially along with increase of the number of the chamber.

(11) $$L_{1}>L_{2}>L_{3}>L_{4}>L_{5}>L_{6}>L_{7}>L_{8}>L_{9}$$

In order to avoid influence of the aperture to the adjustment of length of the cavity, length of the aperture in the proposed APH-AM was set as equal to the thickness of the front panel 2 mm, as shown in the Equation (12). By this method, the adjustable range of length of the cavity could be maximized, which could be more easily to achieve the desired sound absorption performance. Meanwhile, length of the aperture could be enlarged to obtain lower sound absorption performance for the other application conditions.

(12) $$\begin{array}{cc} l_{i}=t_{0}=2 & i=1,2,\ldots ,9 \end{array}$$

Meanwhile, based on the theoretical analysis in the Section 2.2, the other parameters for the proposed APH-AM were set as *d*_i_ =2.7 mm and *a*_i_ = 10 mm (*i* = 1, 2, …, 9). These preset parameters could be adjusted for various application conditions. For example, if the desired frequency range is in the very low frequency region, length of the aperture should be set larger and diameter of the aperture should be smaller, and size of the cavity should be larger as well, which aimed to shift the resonance frequency to low frequency direction.

**Figure 8:**
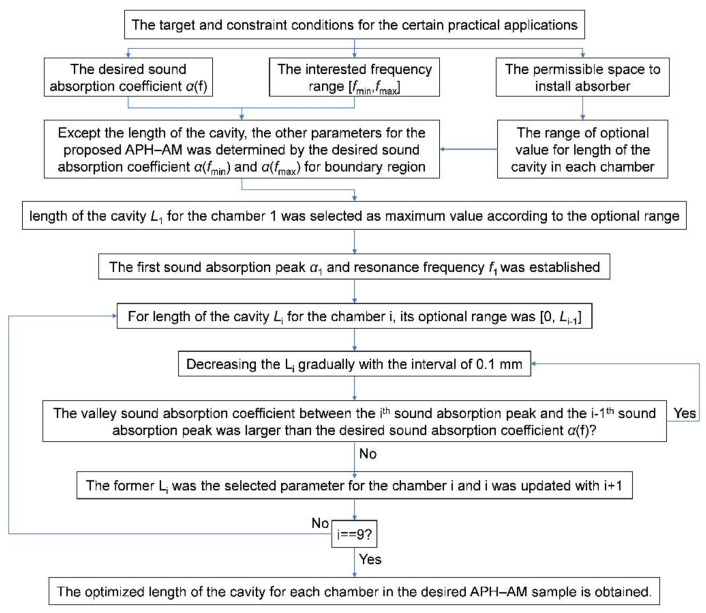
The optimization process in an achievement of the desired sound absorption performance for the APH-AM sample.

Taking the sound absorption requirements of two factory workshops for the targets in this research. The frequency spectrum of noise generated by the equipment in the first factory workshop was [650 Hz, 1250 Hz], and the desired sound absorption coefficients should be larger than 0.9 in this frequency region. The frequency spectrum of noise generated by the equipment in the second factory workshop was [650 Hz, 1450 Hz], and the desired sound absorption coefficients should be larger than 0.85 in this frequency region. Meanwhile, the permissible space to install the acoustic metamaterials was limited to 90 mm. Thus, these two targets were set to investigate effectiveness of the proposed APH-AM. First target was a broad bandwidth with all the sound absorption coefficients above 0.9, and second target was a broad bandwidth with all the sound absorption coefficients above 0.85. It could be judged from the theoretical analysis in Section 2.2 that the resonance frequency *f*_0_ would shift to the low frequency direction along with the increase of length of the cavity *L*, so the length of the cavity *L*_1_ for the chamber 1 was selected as 90 mm according to the optional range and the rule in the Equation (11). Afterwards, through adjusting length of the cavity *L*_2_ for the chamber 2 by controlling the slider moving along the guide in it driven by the linear actuator, the second sound absorption peak was generated, and the optimized parameter was obtained when valley sound absorption coefficient between the first sound absorption peak and the second sound absorption peak was larger than 0.9 for the first target and be larger than 0.85 for the second target. After that, the optimized parameters for chambers 1 and 2 were kept, and the third sound absorption peak was generated through adjusting length of the cavity *L*_3_ for the chamber 3 by controlling the slider moving along the guide in it driven by the linear actuator, and the optimized parameter was obtained when the valley sound absorption coefficient between the second sound absorption peak and the third sound absorption peak was larger than 0.9 for the first target and be larger than 0.85 for second target. Analogously, the optimized parameters for other chambers were obtained. Above all, the whole optimization process was summarized in the Figure 8.

The optimization process shown in the Figure 8 was directly conducted in the experimental measurement instead of the theoretical modeling or finite element simulation, and it would avoid the errors in the constructed theoretical model or the built finite element simulation model, which could improve the accuracy and efficiency in achieving the desired sound absorption performance for the proposed APH-AM.

### 2.4. Transfer Function Tube Measurement

In order to reduce the fabrication cost, the chambers in the APH-AM were fabricated individually with steel pipe with the desired parameters. The guides and slide were fixed into the steel pipe to obtain tunable cavity. All the chambers were fixed together by welding according to the identifiers in the Figure 2a,b. Afterwards, the front panel was processed through the laser drilling of steel panel to gain the required aperture with the desired parameters, and it was fixed to the welded multiple chambers. By these ways, the proposed APH-AM with tunable cavities was prepared.

**Figure 9:**
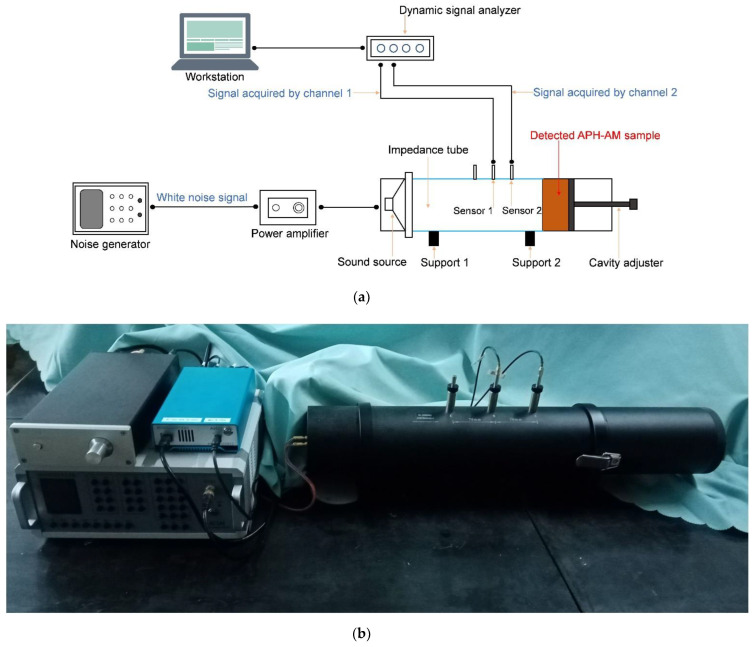
The transfer function tube measurement with detector of AWA6290T. (**a**) A schematic diagram; (**b**) an actual photo.

Experimental detection of the proposed APH-AM was realized by the transfer function tube measurement with the detector of AWA6290T (supported by Hangzhou Aihua Instruments Co., Ltd., Hangzhou, China), as shown in the Figure 9, which could detect the sound absorption coefficients of sound absorbing material or structures with normal incidence according to the GB/T 18696.2-2002 (ISO 10534-2:1998) “Acoustics-Determination of sound absorption coefficient and impedance in impedance tubes-part 2: Transfer function method”. The AWA6290T detector consisted of the AWA5871 power amplifier, the AWA6290B dynamic signal analyzer, the AWA8551 impedance tube, and the corresponding analysis software in the workstation, as shown in the Figure 9. The analysis software could finish the 1/3 OCT analysis and fast Fourier transform (FFT) analysis. Meanwhile, the original incident acoustic wave was also controlled by the signal generation software in the workstation. The detected APH-AM sample was fixed in the end of the impedance tube and two sensors were utilized to detect the signal of the incident and reflected acoustic waves. The distance between sensor 1 and sensor 2 was selected as 70 mm. The detected frequency range was 200–1600 Hz and there was 1502 sampling frequency points in this range. Moreover, for the purpose of elimination of the accidental error, the detection was repeated for 200 times for each sampling frequency point, and the final data was average of the 200 values obtained in the 200 times of measurement. The measurement process was full-automatic, which only took no more than 1 min for once detection program.

Meanwhile, in order to avoid interference with the measurement process, the adjustment of length of the cavity by moving the slider along the guide in chamber driven by the linear actuator was conducted outside, and the well-tuned APH-AM was placed in the AWA6290T detector to measure.

**Figure 10:**
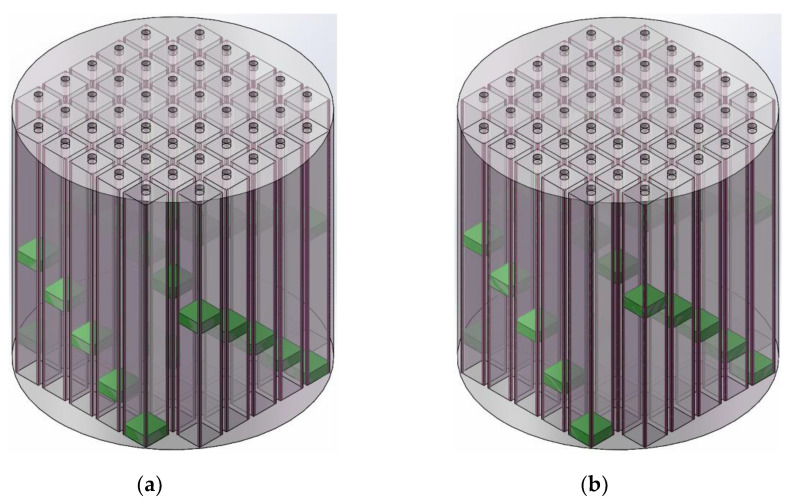

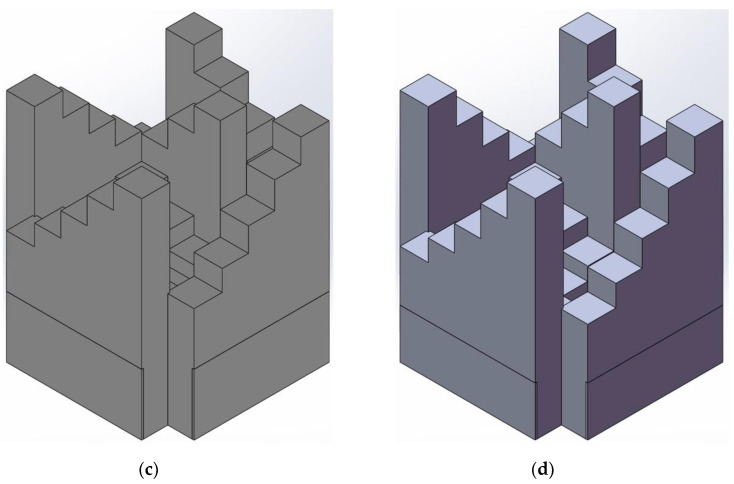
The optimized APH-AM samples. (**a**) A schematic diagram of sample for the target with all the sound absorption coefficients above 0.9; (**b**) a schematic diagram of sample for the target with all the sound absorption coefficients above 0.85. (**c**) the equivalent structure of the sample for the target with all the sound absorption coefficients above 0.9; (**d**) the equivalent structure of the sample for the target with all the sound absorption coefficients above 0.85.

By the multiple optimization process consisted of alternate adjustment and measurement according to the program proposed in the Section 2.3 and exhibited in the Figure 8, the desired APH-AM samples for the two targets was obtained, which were exhibited in the Figure 10, and the optimized length of the cavity for each chamber were summarized in the Table 5. Taking the control accuracy of the length of the cavity into consideration, the values of optimized length of the cavity for each chamber in the 2 desired APH-AM samples were kept one decimal place. It could be found that the minimal length of the cavity for the target of all the sound absorption coefficients above 0.9 was 25.6 mm and that for the target of all the sound absorption coefficients above 0.85 was 20.2 mm. Moreover, its corresponding sound absorption performance was analyzed and the absorption mechanism was investigated by acoustic finite element simulation in the following section.

## 3. Results and Discussions

### 3.1. Actual Sound Absorption Performance

**Figure 11:**
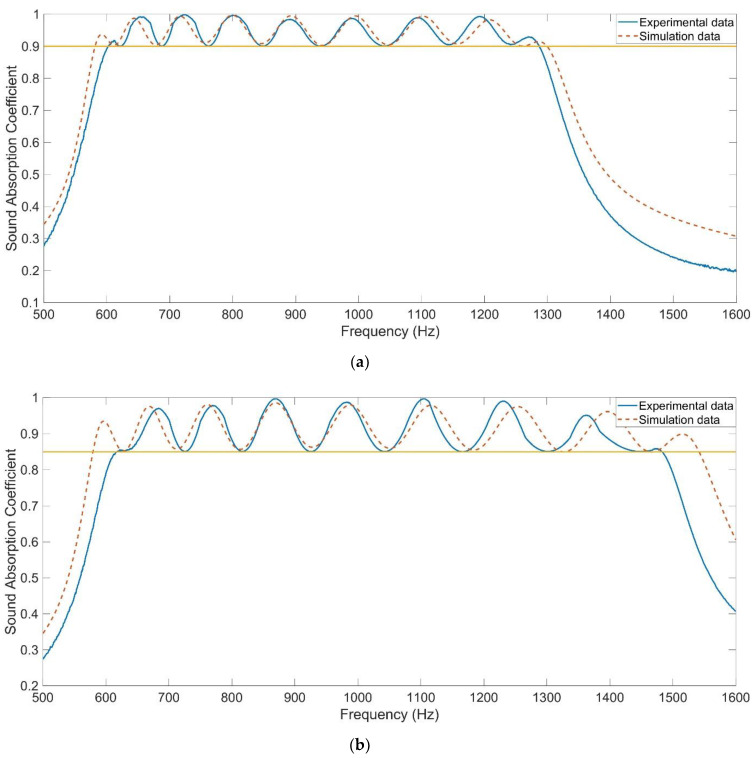
Distributions of sound absorption coefficients of the optimized APH-AM samples in actual and those obtained in finite element simulation. (**a**) For the target with all the sound absorption coefficients above 0.9; (**b**) For the target with all the sound absorption coefficients above 0.85.

Distributions of sound absorption coefficients of the optimized APH-AM samples for the two targets were shown in the Figure 11, both in actual and in simulation. It could be observed that the optimized APH-AM sample could achieve a wide absorption band of 602–1287 Hz with all the sound absorption coefficients above 0.9 and another absorption band of 618–1482 Hz with all the sound absorption coefficient above 0.85, which exhibited excellent sound absorption performance with broad sound absorption width in the low frequency range. Meanwhile, it could be observed that the theoretical sound absorption coefficients obtained by the acoustic finite element simulation were basically consistent with these achieved in experiment, especially for the resonance frequencies for the nine series of chambers. The summaries of resonance frequencies and peak sound absorption coefficients in actual and those in simulation were exhibited in the Table 6. It could be calculated that the deviation for peak sound absorption coefficient *α*_max_ was kept in the range of [−0.08, 0.018] with the absolute value of relative error smaller than 10%, and that for resonance frequency *f*_0_ was kept in the range of [−41.1 Hz, 26.6 Hz] with the absolute value of relative error smaller than 5%. Generally speaking, the sound absorption performance for the target frequency range in simulation was better than that in actual. The major reason for this phenomenon was that there existed errors for the actual structural parameters of APH-AM sample, and the acoustic finite element model was constructed completely consistent with the theoretical parameters. The errors were ineluctable, especially when values of the parameters were small, which indicated that their relative errors were large. These errors would weaken the coupling sound absorption effect for the resonators with the same parameters to generate a better sound absorption peak. Meanwhile, in the actual APH-AM sample, the 4 slides would take some space of chamber, which also resulted in the deviations between the theoretical results and experimental data. With the total thickness of 90 mm, the average sound absorption coefficient of APH-AM sample for target 1 was 0.9441 in the 602–1287 Hz and that for target 2 was 0.9067 in 618–1482 Hz.

Generally speaking, distributions of the sound absorption coefficients in simulation are consistent with those in experiment, because the finite element model simulated the actual measurement process. Meanwhile, it was found from Figure 11 that there existed some deviations between the simulation results and experimental data, especially in the boundary of the target frequency range. It could be also judged from Table 6 that the deviations of resonance frequency *f*_0_ were 19.6 Hz and −15.9 Hz for the first target and those were 26.6 Hz and −41.1 Hz for the second target, which indicated that the achieved satisfactory absorption bandwidth in simulation was larger than that in actual. As mentioned above, the actual satisfactory absorption bands were [602 Hz, 1287 Hz] for the first target and [618 Hz, 1482 Hz] for the second target respectively, which were obviously smaller relative to the theoretical satisfactory absorption bands of [582 Hz, 1298 Hz] for the first target and [580 Hz, 1542 Hz] for the second target obtained in the finite element simulation. The finite element simulation results were basically consistent with the experimental data, and there were obvious existing deviations between satisfactory absorption bandwidth in simulation and that in actual at the same time. It was supposed that these two phenomena were not incompatible. The results obtained by the finite element simulation were mainly consistent with the experimental data, that was the foundation of many optimization processes for the present researches, and the existing deviations should be taken into consideration in the practical applications. Meanwhile, the optimization process of APH-AM in this study could be directly conducted by experimental detection, which was favorable to improve the efficiency and accuracy in achieving the desired sound absorption property.

### 3.2. Sound Absorption Mechanism

**Figure 12:**
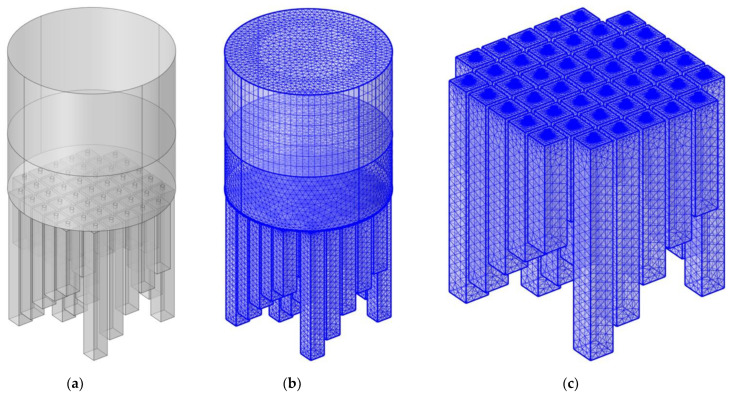

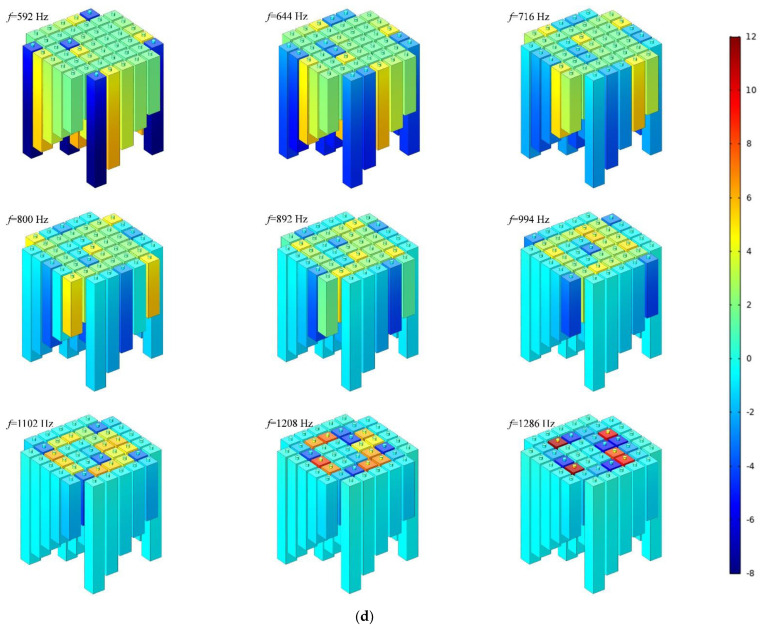

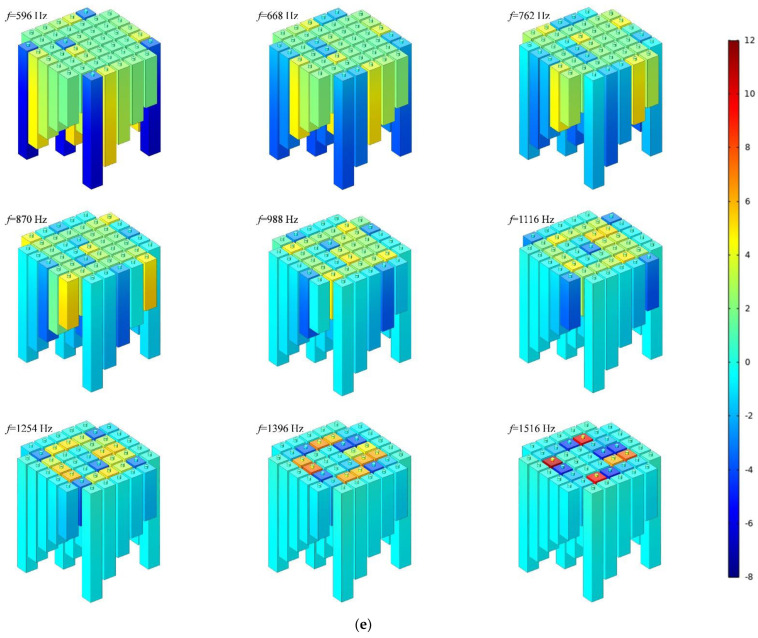
A finite element simulation of the APH-AM. (**a**) The whole 3D structural model; (**b**) the whole gridded model; (**c**) the gridded model of APH-AM; (**d**) distributions of acoustic pressure for the target with all sound absorption coefficients above 0.9; (**e**) distributions of acoustic pressure for the target with all sound absorption coefficients above 0.85.

The sound absorption effect of APH-AM was realized by the resonance effect of the resonators with the same parameters and the coupling effect among the resonators with the different parameters [45,46,47,48], which could be judged from the distribution of acoustic pressure at the resonance frequencies obtained in acoustic finite element simulation models for the two APH-AM samples with normal incidence, as shown in the Figure 12. The whole 3D structural model, the whole gridded model and the gridded model of APH-AM were shown in the Figure 12a–c respectively, which were constructed similarly with the finite element simulation model for the double resonators in the Figure 2. Length of the cavity for each chamber in the APH-AM samples was set according to the parameters in the Table 5. The parameters for the gridded mesh consisted of size of the maximum cell 3.7 mm, size of the minimum cell 0.037 mm, maximum unit growth rate 1.3, curvature factor 0.2, and the resolution of the narrow region 0.95. In order to increase the simulation accuracy, the boundary layers were refined with the stretching factor 1.2 and regulatory factor of their thicknesses 1. Meanwhile, the plane wave with the amplitude 1 was utilized in the background acoustic field as the incident sound wave with normal incidence, and the perfect matching layer was used to simulate the perfect absorption of sound waves in the process of propagation away from the sound source.

It could be found from Figure 12d,e that for each resonance frequency, there was a corresponding group of 5 chambers with the same parameters to generate it. Taking the APH-AM sample for target 1 for the example, the resonance frequencies of 592 Hz, 644 Hz, 716 Hz, 800 Hz, 892 Hz, 994 Hz, 1102 Hz, 1208 Hz and 1286 Hz in the Figure 12d corresponded to the chambers 1 to 9 in the Figure 1b, and this phenomenon was applicable to those resonance frequencies of 596 Hz, 668 Hz, 762 Hz, 870 Hz, 988 Hz, 1116 Hz, 1254 Hz, 1396 Hz and 1516 Hz in the Figure 12e as well. Meanwhile, it could be observed that for a certain resonance frequency, these neighboring chambers around the resonator could also provide some effect to the sound absorption, which could be judged from the color of these chambers in the Figure 12d,e. It should be noted that the “neighbor” here referred to the adjacent parameters instead of the nearby spatial domain. Moreover, the differences between neighboring resonance frequencies in the Figure 12d were 52 Hz, 72 Hz, 84 Hz, 92 Hz, 102 Hz, 108 Hz, 106 Hz and 78 Hz successively, and those in the Figure 12e were 72 Hz, 94 Hz, 108 Hz, 118 Hz, 128 Hz, 138 Hz, 142 Hz and 120 Hz successively, which corresponded to the effective absorption bandwidth for each absorption peak. It could be found that the effective absorption bandwidth of each absorption peak increased at first and then decreased along with increase of the resonance frequency, which indicated the coupling effect was better for the chambers with median parameters relative to those with boundary parameters. Furthermore, it could be found that the first resonance frequency of APH-AM for target 1 was 592 Hz, which was slightly smaller than that 596 Hz of APH-AM for target 2. The major reason for this phenomenon was that the coupling effects of neighboring chambers in the APH-AM decreased with increase of the difference of resonance frequencies.

### 3.3. Sound Absorption Performance with Oblique Incidence

**Figure 13:**
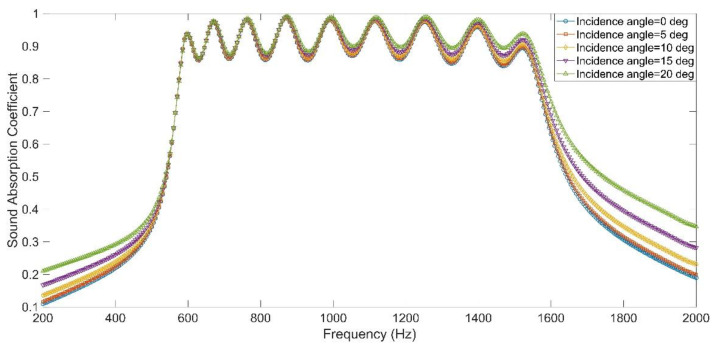
The sound absorption performance of the APH-AM with variable oblique incidence angle obtained in simulation.

Due to the edge diffraction effect, oblique incidence methods considering an infinite sample fail to measure the absorption coefficient at large incidence angles of finite samples [49]. Limited by the utilized transfer function tube detector of AWA6290T, only the sound absorption performance of APH-AM with normal incidence was investigated in the experiment. The sound absorption performance with oblique incidence required the modified detector [50] or the reverberation chamber method [51]. For the classical transfer function tube detector, measurement of sound absorption performance with oblique incidence required the incident tube, the sample, reflected tube with rear perfect absorbing material, and the intersection angle between incident tube and reflected tube should be adjusted to be equal to the oblique incidence angle [50]. Meanwhile, the normal line of detected sample should be consistent with the intersection angle between incident tube and reflected tube [50]. The measurement in the reverberation chamber required a sample with a very large size [51]. In order to reduce the experimental cost, the finite element simulation had been introduced into investigating sound absorption performance with oblique incidence [52]. Meanwhile, it could be found from Figure 11 that the distribution of sound absorption coefficients achieved in simulation was consistent with that obtained in experiment, and the oblique incidence was easy to conduct in the finite element simulation. Therefore, taking the APH-AM sample for the target with all the sound absorption coefficients above 0.85 for example, its sound absorption coefficients under the oblique incidence (the angle was 5–20° with the interval of 5°) were investigated by the finite element simulation, and the results were shown in the Figure 13. The investigated frequency range was 200–2000 Hz and the sampling interval was 5 Hz. It could be judged from Figure 13 that the sound absorption performance was slightly improved along with increase of the incidence angle from 0° to 20°, which was consistent with the similar conclusions obtained in the literatures [49,50,51,52]. Wang et al. [51] had proved that the equivalent acoustic impedance would decrease as the incidence angle increased, which changed the acoustic impedance matching conditions. For the oblique incidence with angle smaller than 45°, change of the sound absorption performance was not obvious, no matter for the resonance frequencies or the peak sound absorption coefficient [51]. In future research, the APH-AM sample would be automatic and adaptive referring to the electronically programmable metasurface.

### 3.4. Sound Absorption Performance with Lower Absorption Band

As known to all, the major advantage of acoustic metamaterial was its effective sound absorption in the low frequency region. Therefore, besides the low frequency sound absorption performance exhibited in the Figure 11, the sound absorption performance of the proposed APH-AM was further studied to achieve lower absorption band. According to the result of influence of the 5 parameters to sound absorption performance of the double resonators, the perforation ratio was restricted by limit of diameter of the aperture in the chamber, and size of the cavity was also difficult to realize for an already prepared acoustic metamaterial, because the change of side length of one chamber would inevitably affect that of the neighboring chambers around it. Therefore, adjustment of length and diameter of the aperture was conducted by finite element simulation, which aimed to achieve lower sound absorption performance. Meanwhile, the APH-AM sample for the target with all the sound absorption coefficients above 0.85 was taken as the example.

#### 3.4.1. Larger Length of the Aperture

**Figure 14:**
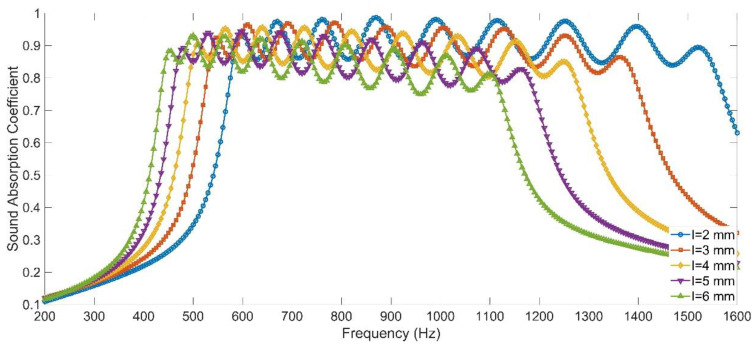
The sound absorption performance of the APH-AM with variable length of the aperture achieved in simulation.

It could be judged from Figure 5 that the increase of length of the aperture could shift the resonance frequencies to the lower frequency direction. Therefore, keeping the other parameters of the APH-AM sample for the target with all sound absorption coefficients above 0.85 in the Table 5 invariant, the sound absorption coefficients were obtained by the finite element simulation with the increase of length of the aperture from 2 mm to 6 mm with the interval of 1 mm, and the simulation results in the frequency range of 200–1600 Hz with the interval of 5 Hz were exhibited in the Figure 14. It could be found that the absorption band shifted to the low frequency direction. When the length of the aperture was 6 mm, all the sound absorption coefficients were larger than 0.75 in the frequency range of 435–1120 Hz and the average sound absorption coefficient in this range reached 0.8391. Meanwhile, this sound absorption performance could be further improved by adjusting the length of the cavity for each chamber. The absorption band could further shift to the lower frequency region by selecting a larger length of the aperture.

#### 3.4.2. Smaller Diameter of the Aperture

**Figure 15:**
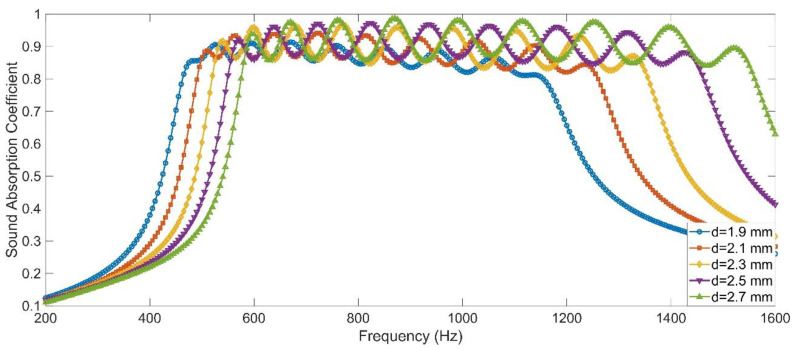
The sound absorption performance of the APH-AM with variable diameter of the aperture gained in simulation.

Similarly, it could be judged from Figure 4 that the decrease of diameter of the aperture could shift the group of resonance frequencies to the low frequency direction. Therefore, keeping the other parameters of the APH-AM sample for the target with all sound absorption coefficients above 0.85 in the Table 5 invariant, the sound absorption coefficients were obtained by the finite element simulation with the decrease of length of the aperture from 2.7 mm to 1.9 mm with the interval of 0.2 mm, and the simulation results in the frequency range of 200–2000 Hz with the interval of 5 Hz were exhibited in the Figure 15. It could be observed that when the diameter of the aperture was 1.9 mm, all the sound absorption coefficients were larger than 0.75 in the frequency range of 455–1175 Hz and the average sound absorption coefficient in this range reached 0.8602. Similarly, this sound absorption performance could also be further improved by adjusting the length of the cavity for each chamber. Meanwhile, the sound absorption band could further shift to the lower frequency region by selecting smaller diameter of the aperture.

Increase of length of the aperture and decrease of diameter of the aperture could be conducted simultaneously for the proposed APH-AM, which could obtained a lower frequency band. For the certain target sound absorption requirement, diameter and length of the aperture and size and maximal length of the cavity could be established, and the suitable length of the cavity for each chamber in the APH-AM could be achieved in the transfer matrix tube measurement according to the optimization process in the Section 2.2.

## 4. Conclusions

The major achievements obtained in this study were as follows:

(1) An APH-AM was proposed and developed by introducing the multiple resonant chambers and tuning the length of rear cavity for each chamber, and its sound absorption performance could be easily adjusted by changing length of the cavity by moving the slide along the guides in each chamber.
(2) A 3-dimensional finite element simulation model for the double resonators was constructed to analyze influence of parameters to sound absorption performance of APH-AM, which provide guidance to adjust the APH-AM sample.
(3) With the total thickness of 90 mm for the APH-AM sample, the target for each sound absorption coefficient above 0.9 was achieved in the frequency range of 602–1287 Hz, and that for each sound absorption coefficient above 0.85 was obtained in the 618–1482 Hz. Meanwhile, the average sound absorption coefficient of the APH-AM sample for target 1 was 0.9441 in the 602–1287 Hz and that for target 2 was 0.9067 in the 618–1482 Hz, which exhibited an outstanding sound absorption performance.
(4) The sound absorption mechanism of the APH-AM was studied by calculating the distribution of acoustic pressure in the finite element simulation, which would be propitious to promote the practical application of the APH-AM and similar acoustic materials.
(5) Through increasing length of the aperture or decreasing diameter of the aperture, sound absorption performance with the lower frequency region was achieved in the finite element simulation, which could further exhibit advantages of the proposed APH-AM sample in absorbing the noise in the low frequency region.

## Figures and Tables

**Table 1:** A summary of the peak sound absorption coefficient *α*_max_ and resonance frequency *f*_0_ for double resonators with different diameter of the aperture.

Parameters		First Absorption Peak		Second Absorption Peak	
*d*_1_	*d*_2_	*f*_0_	*α*_max_	*f*_0_	*α*_max_
2.7 mm	1.7 mm	610 Hz	0.84	404 Hz	0.98
2.7 mm	2.2 mm	610 Hz	0.97	520 Hz	0.99
2.7 mm	2.7 mm	618 Hz	0.92	618 Hz	0.92
2.7 mm	3.2 mm	618 Hz	0.95	710 Hz	0.99
2.7 mm	3.7 mm	616 Hz	0.87	804 Hz	0.99

**Table 2:** The summary of the peak sound absorption coefficient *α*_max_ and resonance frequency *f*_0_ for double resonators with different length of the aperture.

Parameters		First Absorption Peak		Second Absorption Peak	
*l*_1_	*l*_2_	*f*_0_	*α*_max_	*f*_0_	*α*_max_
6 mm	2 mm	619 Hz	0.99	826 Hz	0.96
6 mm	4 mm	625 Hz	0.99	691 Hz	0.99
6 mm	6 mm	618 Hz	0.92	618 Hz	0.92
6 mm	8 mm	610 Hz	0.99	565 Hz	0.98
6 mm	10 mm	613 Hz	0.99	517 Hz	0.96

**Table 3:** The summary of the peak sound absorption coefficient *α*_max_ and resonance frequency *f*_0_ for double resonators with different length of the cavity.

Parameters		First Absorption Peak		Second Absorption Peak	
*L*_1_	*L*_2_	*f*_0_	*α*_max_	*f*_0_	*α*_max_
50 mm	30 mm	618 Hz	0.99	819 Hz	0.99
50 mm	40 mm	621 Hz	0.99	693 Hz	0.99
50 mm	50 mm	618 Hz	0.92	618 Hz	0.92
50 mm	60 mm	615 Hz	0.99	564 Hz	0.99
50 mm	70 mm	615 Hz	0.99	510 Hz	0.99

**Table 4:** The summary of the peak sound absorption coefficient *α*_max_ and resonance frequency *f*_0_ for double resonators with different size of the cavity.

Parameters		First Absorption Peak		Second Absorption Peak	
*a*_1_	*a*_2_	*f*_0_	*α*_max_	*f*_0_	*α*_max_
10 mm	6 mm	618 Hz	0.99	974 Hz	0.95
10 mm	8 mm	620 Hz	0.99	756 Hz	0.99
10 mm	10 mm	618 Hz	0.92	618 Hz	0.92
10 mm	12 mm	612 Hz	0.99	522 Hz	0.99
10 mm	14 mm	614 Hz	0.99	446 Hz	0.99

**Table 5:** The summary of the optimized length of the cavity for each chamber in the desired APH-AM samples.

Target	Parameters (mm)								
	*L*_1_	*L*_2_	*L*_3_	*L*_4_	*L*_5_	*L*_6_	*L*_7_	*L*_8_	*L*_9_
Each *α* above 0.9	90	79.2	67.2	56.5	47.2	39.7	33.4	28.6	25.6
Each *α* above 0.85	90	75.9	62.2	50.7	41.1	33.9	27.9	23.2	20.2

**Table 6:** The summary of the peak sound absorption coefficient *α*_max_ and resonance frequency *f*_0_ in actual and those in simulation for the optimized APH-AM samples.

	Serial Number of Sound Absorption Peak	Peak Sound Absorption Coefficient *α*_max_			Resonance Frequency *f*_0_		
		In Actual	In Simulation	Deviation	In Actual	In Simulation	Deviation
Target with *α* above 0.9	1	0.9185	0.9375	−0.0190	611.6	592	19.6
	2	0.9908	0.9874	0.0034	651.1	644	7.1
	3	0.9983	0.9943	0.0040	723.6	716	7.6
	4	0.9966	0.9974	−0.0008	800.5	800	0.5
	5	0.9835	0.9952	−0.0117	889.9	892	−2.1
	6	0.9869	0.9947	−0.0078	988.8	994	−5.2
	7	0.9894	0.9937	−0.0043	1094.2	1102	−7.8
	8	0.9929	0.9826	0.0103	1192.3	1208	−15.7
	9	0.9294	0.9145	0.0149	1270.1	1286	−15.9
Target with *α* above 0.85	1	0.8552	0.9348	−0.0796	622.6	596	26.6
	2	0.9702	0.9758	−0.0056	684.1	668	16.1
	3	0.9779	0.9809	−0.0030	769.8	762	7.8
	4	0.9967	0.9858	0.0109	870.1	870	0.1
	5	0.9873	0.9801	0.0072	982.2	988	−5.8
	6	0.9965	0.9788	0.0177	1106.8	1116	−9.2
	7	0.9903	0.9754	0.0149	1232.2	1254	−21.8
	8	0.9512	0.9613	−0.0101	1362.1	1396	−33.9
	9	0.8585	0.8987	−0.0402	1474.9	1516	−41.1

## Data Availability

The data that support the findings of this study are available from the corresponding author upon reasonable request.
